# Occurrence of extrapulmonary tuberculosis is associated with geographical origin: spatial characteristics of the Frankfurt TB cohort 2013–2018

**DOI:** 10.1007/s15010-022-01921-9

**Published:** 2022-10-01

**Authors:** Nils Wetzstein, Alena-Pauline Drummer, Annabelle Bockey, Eva Herrmann, Claus Philippe Küpper-Tetzel, Christiana Graf, Benjamin Koch, Udo Goetsch, Maria J. G. T. Vehreschild, Lorenzo Guglielmetti, Berit Lange, Thomas A. Wichelhaus, Christoph Stephan

**Affiliations:** 1 Department of Internal Medicine, Infectious Diseases, University Hospital Frankfurt, Goethe University, Frankfurt am Main, Germany; 2 Department for Epidemiology, Helmholtz Centre for Infection Research, Braunschweig, Germany; 3 Institute of Biostatistics and Mathematical Modeling, Goethe University, Frankfurt am Main, Germany; 4 Department of Internal Medicine, Gastroenterology and Hepatology, University Hospital Frankfurt, Goethe University, Frankfurt am Main, Germany; 5 Department of Internal Medicine, Nephrology, University Hospital Frankfurt, Goethe University, Frankfurt am Main, Germany; 6 Health Protection Authority, City of Frankfurt, Frankfurt am Main, Germany; 7 Laboratoire de Bactériologie-Hygiène, Centre National de Référence des Mycobactéries et de la Résistance des Mycobactéries aux Antituberculeux, APHP, Groupe Hospitalier Universitaire Sorbonne Université, Hôpital Pitié-Salpêtrière, Paris, France; 8 Sorbonne Université, INSERM, U1135, Centre d’Immunologie et des Maladies Infectieuses, Cimi-Paris, Paris, France; 9 Institute of Medical Microbiology and Infection Control, University Hospital Frankfurt, Goethe University, Frankfurt am Main, Germany

**Keywords:** Tuberculosis, TB, *Mycobacterium tuberculosis* complex

## Abstract

**Introduction:**

Tuberculosis (TB) is caused by *M. tuberculosis* complex (MTB) and pulmonary tuberculosis (PTB) is its classical manifestation. However, in some regions of the world, extrapulmonary TB (EPTB) seems to be more frequent.

**Methods:**

We performed a retrospective cohort study of all TB patients treated at University Hospital Frankfurt, Germany, for the time period 2013–2018. Patient charts were reviewed and demographic, clinical, and microbiological data recorded. Patients were subdivided according to their geographic origins.

**Results:**

Of the 378 included patients, 309 were born outside Germany (81.7%). Three WHO regions were significantly associated with the occurrence of isolated EPTB: the South-East Asian Region (OR 3.37, CI 1.74–6.66, *p* < 0.001), the African Region (2.20, CI 1.25–3.90, *p* = 0.006), and the Eastern Mediterranean Region (OR 3.18, CI 1.78–5.76, *p* < 0.001). On a country level, seven countries of origin could be demonstrated to be significantly associated with the occurrence of isolated EPTB: India (OR 5.58, CI 2.30–14.20, *p* < 0.001), Nepal (OR 12.75, CI 1.73–259.28, *p* = 0.027), Afghanistan (OR 3.64, CI 1.14–11.98, *p* = 0.029), Pakistan (OR 3.64, CI 1.14–11.98, *p* = 0.029), Eritrea (OR 3.32, CI 1.52–7.47, *p* = 0.003), Somalia (OR 7.08, CI 2.77–19.43, *p* < 0.001), and Turkey (OR 9.56, CI 2.52–47.19, *p* = 0.002).

**Conclusion:**

Geographical origin is a predictor for the occurrence of extrapulmonary TB. This might be linked to a delay in diagnosis in these patients, as well as specific responsible impairments of the host’s immune system, possible virulence factors of MTB, and relevant comorbidities.

**Supplementary Information:**

The online version contains supplementary material available at 10.1007/s15010-022-01921-9.

## Introduction

Tuberculosis (TB) is a multi-systemic disease caused by the *M. tuberculosis* complex (MTB, comprising among others *M. tuberculosis*, *M. bovis*, and *M. africanum*) [1]. It is presumed that approximately 1.7 billion individuals are at least latently infected with MTB [2]. However, only a fraction of those patients develop clinically symptomatic disease within their lifetimes [3]. The Global Tuberculosis Report of 2021 estimates that there were approximately 10 million new TB cases for the year 2020. Of those, 3–4% of patients suffer from multidrug-resistant TB or rifampicin-resistant TB (MDR/RR-TB). Eight high incidence countries (India, Pakistan, Nigeria, South Africa, China, Bangladesh, Indonesia, and the Philippines) account for two-thirds of all notified cases worldwide [4]. In high-income countries, such as Germany, TB has become mainly an imported infection among foreign-born patients [5].

TB can be described as a clinical spectrum ranging from latent tuberculous infection (LTBI) to active disease [6]. Pulmonary TB (PTB) is the most common and classical manifestation of TB. On the other hand, there is a large variety of extrapulmonary TB (EPTB) such as tuberculosis of the pleura, lymph nodes, bone, the central nervous system or the genitourinary tract [7]. There seem to be differences in host susceptibility to developing these different clinical manifestations. Recently, an association between the occurrence of extrapulmonary TB with different geographical origins has been described: Sotgiu et al. found an African origin or being from the Indian Subcontinent to be positive predictors for extrapulmonary TB, while Hayward et al. showed that migrants from South-East Asia and sub-Saharan Africa tend to suffer more frequently from extrapulmonary TB (EPTB) than those from other geographic regions [8, 9]. In addition, impairments of cellular immunity (such as HIV or immunosuppressive treatment), as well as specific defects in the interferon-gamma axis (e.g., Mendelian susceptibility to mycobacterial disease) are known risk factors for mycobacterial infections and lead to disseminated infections that might require prolonged antimycobacterial treatment [10]**.**

At Frankfurt University Hospital, we take care of a large number of TB patients in the Metropolitan Region. This group involves, among others, patients with a migration background, patients with HIV infection, as well as immunocompromised patients, for example after solid organ or stem cell transplantation. In this study, we aimed at elucidating whether geographic origin is a predictor for different clinical forms of TB in our cohort.

## Methods

### Database query, inclusion criteria, and exclusion criteria

We performed a laboratory database query for all patients with positive cultures or PCR tests for MTB, as well as a patient database query for all patients coded with ICD-codes A15–A19 for the time period 2013–2018. Patients, for whom no clinical data were available, were excluded, as well as patients that had established the initial diagnosis of TB prior to the observation period. Therefore, we included all patients with a clinical, microbiological, or histological diagnosis of TB from 2013 to 2018.

Using the local hospital patient information system (ORBIS, Agfa Health Care, Bonn, Germany), we performed a chart review to retrieve relevant patient information: age, gender, geographical origin (by country and by WHO regions), education, microbiological results (mycobacterial species, culture, PCR results, susceptibility testing and site of infection), antimycobacterial therapies, clinical manifestations, comorbidities, and the occurrence of lethal events. Observation time was recorded as time from TB diagnosis to the last clinical contact. Data collection forms were adapted from an ongoing TBnet study (https://www.tbnet.eu/migrant-project).

Resistance classifications were applied following the WHO definitions (before 2018) and German guideline definitions [11]: fully drug susceptible (DS-TB), monoresistance (resistance to one first-line TB drug), multidrug-resistant TB (MDR, resistance to isoniazid and rifampicin), extensively drug-resistant TB (XDR, resistance to isoniazid, rifampicin, to at least one fluoroquinolone and one of the injectables), and polyresistance (resistance to more than one first-line TB drug, but not meeting MDR or XDR definitions).

PTB was defined as affection of the lung parenchyma with positive radiological signs or positive microbiological specimens from a respiratory sample. EPTB included all other sites of infections including pleura, lymph nodes, abdominal manifestation, bone affection, urogenitary TB, and affection of the central nervous system, the spine, or others. This study was approved by our local ethics committee under file number 2021–270.

### Statistical analysis

All data were analyzed in R v. 4.1.2 “Bird Hippie” [12]. Continuous data are depicted as mean with range for normally distributed data and as median with interquartile range (IQR) for non-normally distributed data. Categorical variables are shown as numbers and percentage. We used the Wilcoxon signed-rank test to detect differences in continuous data and the Fisher exact for differences in categorical variables between groups. Univariate logistic regression was conducted in R using a linear model. First, all WHO regions were tested against the European Region as a reference. Second, all countries of origin were tested against a German origin as a reference. Multivariate analysis was performed including the geographic origins of patients (in form of the WHO region), HIV status, administration of immunosuppressive therapies, and age. Odds ratios (OR), as well as confidence intervals (CI), were recorded. For all statistical tests, a confidence level of alpha = 0.05 was used. All graphs were drawn using the *ggplot2* package within the *tidyverse* [13, 14].

## Results

### General characteristics

In total, we included 378 patients during the observation period (Fig. S1, Table 1). Most patients were born outside Germany (*n* = 309, 81.7%).

**Table 1:** General characteristics of the included patients

	All	
	(*n* = 378)	
	*n*	[%]
Gender		
Male	220	58.2
Female	158	41.8
Born outside Germany		
Yes	309	81.7
No	69	18.3
Language barrier		
Yes	120	31.7
No	170	45.0
Unknown	88	23.3
Housing		
Own flat/house	217	57.4
Communal accommodation	36	9.5
Social housing	7	1.9
Homeless	9	2.4
Not known	88	23.3
Length in country		
< 1 year	52	13.8
1–3 years	46	12.2
> 3 years	131	34.7
Unknown	80	21.2
NA	69	18.3
Comorbidities		
HIV	47	12.4
Immunosuppressives	33	8.7
Diabetes	35	9.3
Malignancy	27	7.1
CVD	74	19.6
Smoker	57	15.1
CKD	17	4.5
Vitamin D deficiency	62/69	89.9
Outcome		
Deceased	13	3.4

*HIV* human immunodeficiency virus, *CVD* chronic vascular disease, *CKD* chronic kidney disease

A majority of patients were male (*n* = 220, 58.2%) and the median age was 35 years (IQR 29–49 years). 120 patients had a documented relevant language barrier (31.7%), and 42.4% of patients born abroad had been living in Germany for more than 3 years (*n* = 131). Most patients lived in their own flats (*n* = 217, 57.4%), whereas 36 (9.5%) were living in communal accommodation at the time of diagnosis.

47 (12.4%) patients suffered from HIV, 33 (8.7%) were under immunosuppressive therapy, 35 (9.3%) had diabetes, 27 (7.1%) a known malignancy, 74 patients a chronic vascular disease (CVD, including arterial hypertension, 19.6%), 57 (15.1%) were smokers, and 17 (4.5%) suffered from chronic kidney disease (CKD). In patients, in which serum concentration of vitamin D was determined (*n* = 69), 62 (89.9%) suffered from manifest vitamin D deficiency.

Overall, 307 (81.2%) patients had a microbiologically confirmed diagnosis of TB (positive mycobacteriological cultures or PCR for MTB). Of those, 276 (89.9%) were specified as *M. tuberculosis*, 26 (8.5%) only to a complex level (MTB), three (1.0%) were identified as *M. africanum* and two (0.7%) as *M. bovis* (Table 2). The majority of isolates was fully drug susceptible (DS-TB, *n* = 243, 79.2%), 24 isolates were mono-resistant (7.8%), 9 patients suffered from MDR-TB (2.4%) and 4 patients from XDR-TB (1.3%). Nine patients had an isolate with a polyresistance (2.4%).

**Table 2:** Microbiological results and drug susceptibility in isolates from included patients

	All	
	(*n* = 378)	
	*n*	[%]
Microbiologically confirmed diagnosis	307	81.2
No microbiological detection	71	18.8
Mycobacterial species		
* M. tuberculosis*	276	89.9
M. tuberculosis complex	26	8.5
* M. africanum*	3	1.0
* M. bovis*	2	0.7
Drug resistance		
Fully drug susceptible	243	79.2
Monoresistance	24	7.8
Isoniazid	12	3.9
Rifampicin	5	1.6
Pyrazinamide	1	0.3
Streptomycin	8	2.6
Polyresistance	9	2.9
Isoniazid and Streptomycin	6	2.0
Isoniazid and Pyrazinamide	1	0.3
Isoniazid and Prothionamide	1	0.3
Isoniazid, Protionamide and FQ	1	0.3
MDR	9	2.9
XDR	4	1.3

*FQ* fluoroquinolone, *MDR* multidrug resistant, *XDR* extensively drug resistant

### Clinical manifestations, antimycobacterial therapy and outcome

211 patients suffered from PTB (55.8%). However, only a limited fraction of patients had PTB as their only clinical manifestation (*n* = 58, 15.3%) (Table 3). The most frequent site of manifestation of extrapulmonary TB were lymph nodes in 60.1% of patients (*n* = 227), followed by abdominal TB in 19.3% of patients (*n* = 73).

**Table 3:** Frequency of clinical manifestations in included patients

	All	Isolated PTB	Isolated EPTB	Both
	*n* (%)	*n* (%)	*n* (%)	*n* (%)
Total number of patients	378 (100)	58 (15.3)	167 (44.2)	153 (40.5)
Specific organ manifestations				
Pulmonary	211 (55.8)	58 (100)	–	153 (100)
Extrapulmonary	320 (84.7)	–	167 (100)	153 (100)
Pleura	47 (12.4)	–	13 (7.7)	34 (22.2)
Lymph node	227 (60.1)	–	109 (65.3)	118 (77.1)
Abdominal	73 (19.3)	–	27 (16.2)	46 (30.1)
Bone (other than spine)	21 (5.6)	–	16 (9.6)	5 (3.3)
Urogenital	16 (4.2)	–	12 (7.2)	4 (2.6)
CNS	21 (5.6)	–	7 (4.2)	14 (9.2)
Spine	37 (9.8)	–	27 (16.2)	10 (6.5)
Other	44 (11.6)	–	26 (15.6)	18 (11.8)
≥ 2 Extrapulmonary organs involved	133 (35.2)	–	59 (35.3)	74 (38.4)

*PTB* pulmonary TB, *EPTB* extrapulmonary TB

337 patients received a standard TB treatment for DS-TB including isoniazid, rifampicin, pyrazinamide, and ethambutol (89.2%). Overall, 237 patients suffered from adverse events: 155 had elevated liver enzymes (41.0%), 86 (22.8%) had gastrointestinal side effects, and 54 (14.3%) suffered from arthralgia. Other side effects were less frequently reported. In 124 of these patients (32.8%), guideline therapy had to be discontinued.

The median observation time was 408 days (IQR: 165–758, range: 1–3508). We observed an overall case fatality rate of 3.4% (*n* = 13). Median time to death was 28 days (IQR: 7–108, range: 2–428) from the day of diagnosis. Deceased patients had a median age of 51 years (IQR 35–64 years) and 11/13 (84.6%) had at least one relevant comorbidity: 3 were HIV positive, 3 were under immunosuppressive therapy, three suffered from diabetes, 4 patients from malignancy, 8 from CVD, and 3 from CKD.

### Geographical origin and association with different clinical forms of TB

**Fig. 1:**
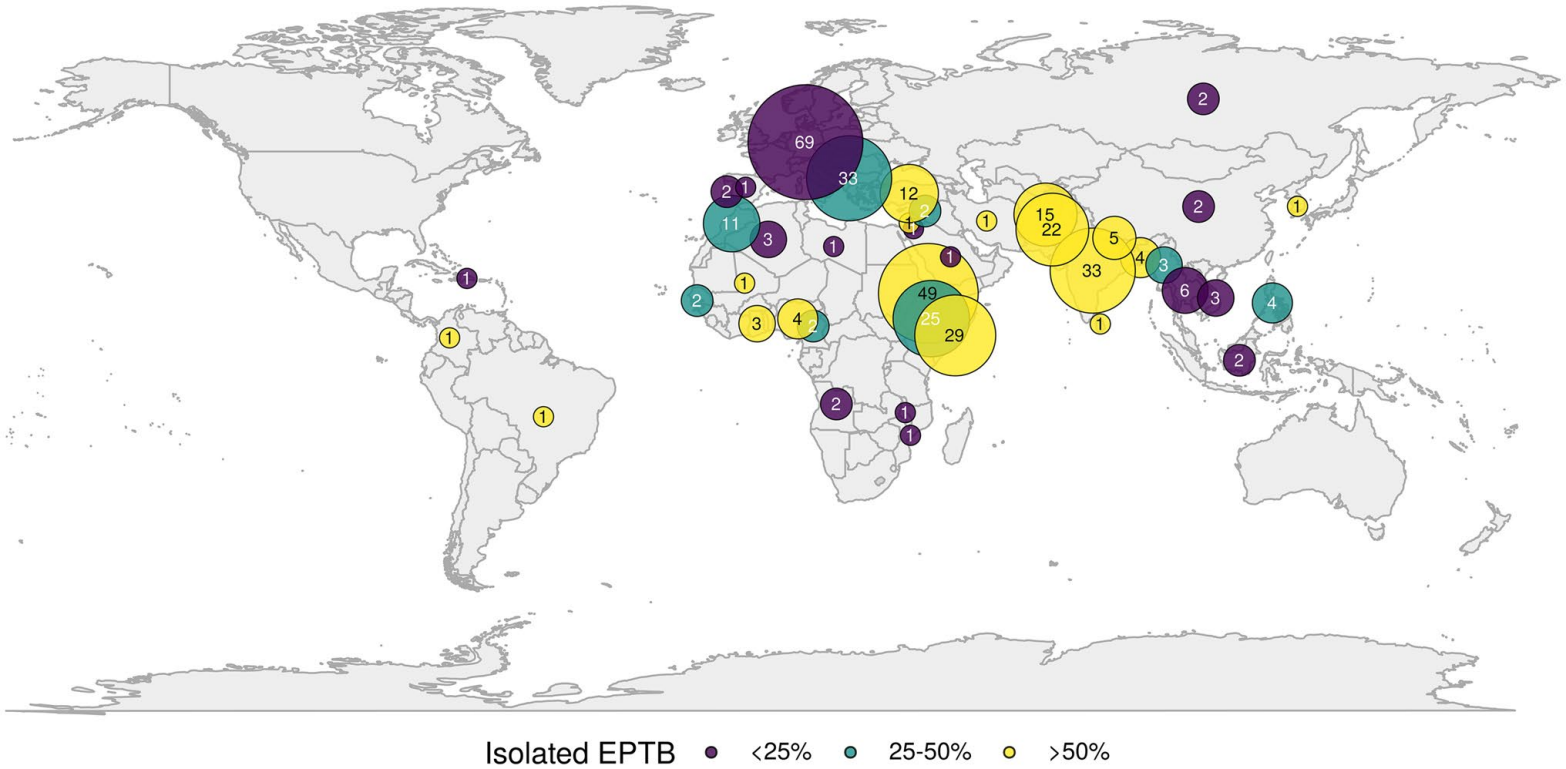
Geographical distribution of included patients (world map) and frequency of isolated extrapulmonary TB (EPTB). The map shows 363 patients for whom migrational status and geographical origin are known. 69 patients were born in Germany or there was no hint of migration in their patient history. Patients from Eastern Europe (Austria, Bosnia, Bulgaria, Croatia, Kosovo, Poland, Romania, Serbia, Ukraine, former Yugoslavia and “Eastern Europe” not further specified) are summarized in the world map

Of the included patients, 94 patients originated from the WHO African Region (AFR, 24.9%), 84 from the Eastern Mediterranean Region (EMR, 22.2%), 54 from the South-East Asian Region (SEAR, 14.3%), 119 from the European Region (including Germany, EUR, 31.5%), 10 from the Western Pacific Region (WPR, 2.6%), and only 3 patients from the Region of the Americas (AMR, 0.8%) (Table 4, Fig. 1). In 14 patients, the region of origin was unknown.

**Table 4:** Geographical origins, clinical manifestations, and respective odds ratios for the occurrence of isolated extrapulmonary TB

	Number of patients	Isolated PTB	PTB and EPTB	Isolated EPTB	OR (CI)*	*p* value*
South-East Asian Region (SEAR)	**54**	**6**	**17**	**31**	**3.37 (1.74–6.66)**	** < 0.001**
Bangladesh	4	0	1	3	9.56 (1.14–200.93)	0.058
India	33	2	10	21	**5.58 (2.30–14.20)**	**0.001**
Indonesia	2	1	1	0	–	–
Myanmar	3	1	1	1	1.59 (0.07–17.72)	0.711
Nepal	5	0	1	4	**12.75 (1.73–259.28)**	**0.027**
Sri Lanka	1	0	0	1	–	–
Thailand	6	2	3	1	0.80 (0.04–5.89)	0.844
African Region (AFR)	**94**	**7**	**43**	**44**	**2.20 (1.25–3.90)**	**0.006**
Algeria	3	1	2	0	0.00 (NA–Inf)	0.994
Angola	2	2	0	0	–	–
Eritrea	49	4	20	25	**3.32 (1.52–7.47)**	**0.003**
Ethiopia	25	0	14	11	2.50 (0.94–6.65)	0.063
Gambia	2	0	1	1	–	–
Ghana	3	0	1	2	6.38 (0.57–142.61)	0.141
Cameroon	2	0	1	1	–	–
Malawi	1	0	1	0	–	–
Mali	1	0	0	1	–	–
Mosambique	1	0	1	0	–	–
Nigeria	4	0	1	3	9.56 (1.14–200.93)	0.058
“Sub-Saharan Africa” (not further specified)	1	0	1	0	–	–
European Region (EUR)	**119**	**28**	**55**	**34**	**–**	**–**
Austria	1	0	1	0	–	–
Bosnien	2	1	0	1	–	–
Bulgaria	3	1	2	0	0.00 (NA–Inf)	0.994
Croatia	7	1	4	2	1.28 (0.17–6.57)	0.784
Germany	69	22	29	16	–	–
Kosovo	3	1	1	1	1.59 (0.07–17.72)	0.711
Poland	1	0	1	0	–	–
Portugal	2	0	2	0	–	–
Romania	8	0	6	2	1.06 (0.15–5.16)	0.944
Russia	2	1	1	0	–	–
Serbia	4	0	2	2	3.19 (0.36–28.36)	0.265
Spain	1	1	0	0	–	–
Turkey	12	0	3	9	**9.56 (2.52–47.19)**	**0.002**
Ukraine	2	0	1	1	–	–
Yugoslavia	1	0	1	0	–	–
"Eastern Europe" (not further specified)	1	0	1	0	–	–
Region of the Americas (AMR)	**3**	**0**	**1**	**2**	5.00 (0.46–109.69)	0.195
Brasil	1	0	0	1	–	–
Colombia	1	0	0	1	–	–
Dominican Republic	1	0	1	0	–	–
Eastern Mediterranean Region (EMR)	**84**	**10**	**27**	**47**	**3.18 (1.78–5.76)**	** < 0.001**
Afghanistan	15	3	4	8	**3.64 (1.14–11.98)**	**0.029**
Iran	1	0	0	1	–	–
Jordan	1	0	1	0	–	–
Libya	1	1	0	0	–	–
Morocco	11	4	4	3	1.20 (0.24–4.71)	0.808
Pakistan	22	2	7	13	**4.60 (1.69–13.17)**	**0.003**
Palestine	1	0	0	1	–	–
Saudi Arabia	1	0	1	0	–	–
Somalia	29	0	9	20	**7.08 (2.77–19.43)**	** < 0.001**
Syria	2	0	1	1	–	–
Western Pacific Region (WPR)	**10**	**3**	**4**	**3**	1.07 (0.22–4.10)	0.924
China	2	1	1	0	–	–
Philippines	4	2	0	2	3.19 (0.36–28.36)	0.265
South Korea	1	0	0	1	–	–
Vietnam	3	0	3	0	0.00 (NA–Inf)	0.994
Unknown	14	4	6	6	NA	NA
All patients	378	58	153	167		

*PTB* pulmonary TB, *EPTB* extrapulmonary TB, *OR* odds ratio, *CI* confidence interval, *NA* not applicable, *Inf* infinite

*For the occurrence of isolated EPTB. Significant associations are shown in bold. ORs and p values for countries with less than three patients are not shown

In the univariate analysis, three WHO regions were significantly associated with the occurrence of isolated EPTB: SEAR (OR 3.37, CI 1.74–6.66, *p* < 0.001), AFR (2.20, CI 1.25–3.90, *p* = 0.006), and the EMR (OR 3.18, CI 1.78–5.76, *p* < 0.001). On a country level, seven countries of origin could be demonstrated to be significantly associated with the occurrence of isolated EPTB: India (OR 5.58, CI 2.30–14.20, *p* < 0.001), Nepal (OR 12.75, CI 1.73–259.28, *p* = 0.027), Afghanistan (OR 3.64, CI 1.14–11.98, *p* = 0.029), Pakistan (OR 3.64, CI 1.14–11.98, *p* = 0.029), Eritrea (OR 3.32, CI 1.52–7.47, *p* = 0.003), Somalia (OR 7.08, CI 2.77–19.43, *p* < 0.001), and Turkey (OR 9.56, CI 2.52–47.19, *p* = 0.002). On the other hand, Ethiopia was the only country with more than 11 patients that did not reach significance for the association with isolated EPTB, but showed only a trend toward it (OR 2.50, CI 0.94–6.65, *p* = 0.063).

**Fig. 2:**
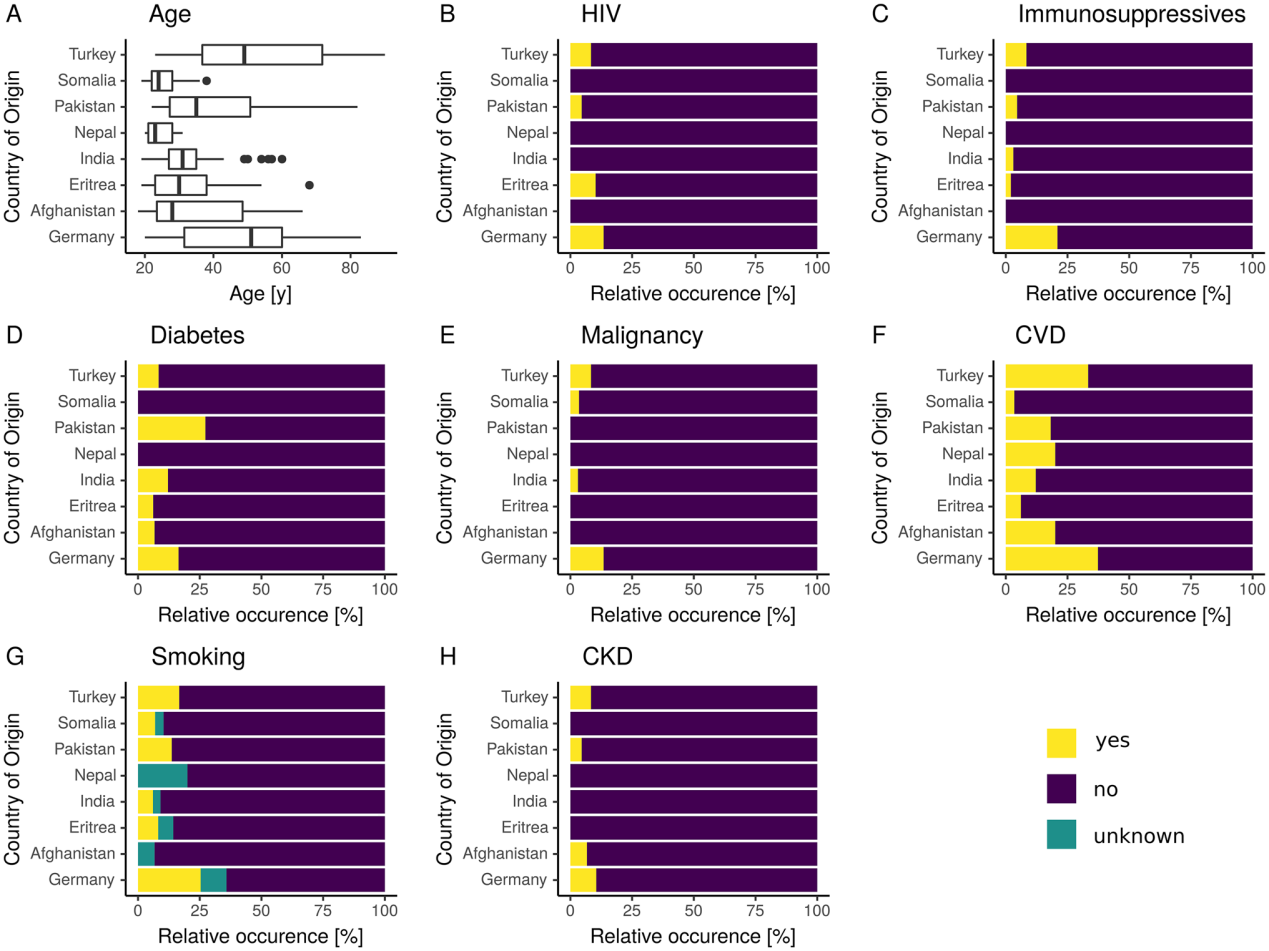
Box plots of age (**A**) and bar graphs of comorbidities (**B**–**H**) in German patients and foreign-born patients whose origin was significantly associated with the occurrence of isolated EPTB (Turkey, Somalia, Pakistan, Nepal, India, Eritrea, and Afghanistan)

Patients from these seven countries were significantly younger than German patients (median 29 years IQR 24–39 vs. 51 years IQR 31.5–60, *p* < 0.001, Fig. 2A), suffered less frequently from HIV (4.2% vs. 13.0%, *p* = 0.02, Fig. 2B), received less immunosuppressive therapies (2.4% vs. 20.9%, *p* < 0.001, Fig. 2C), and suffered less frequently from diabetes (9.1% vs. 16.4%, *p* = 0.11, Fig. 2D), malignoma (1.8% vs. 9.0%, *p* < 0.001, Fig. 2E), CVD (12.1% vs. 38.4%, *p* < 0.001, Fig. 2F), tobacco addiction (7.9% vs. 26.2%, *p* < 0.001, Fig. 2G), and CKD (1.8% vs. 10.4%, *p* < 0.01, Fig. 2H)*.*

Multivariate analysis showed the same geographic regions to be associated with the occurrence of isolated extrapulmonary TB (Table 5). On the other hand, an HIV infection or an immunosuppressive therapy was a negative predictor (OR 0.25, CI 0.10–0.55, *p* = 0.001, and OR 0.20, CI 0.06–0.54, *p* = 0.004, respectively).

**Table 5:** Multivariate analysis including the WHO region, HIV status, the reception of immunosuppressive therapies, and age as predictors for the occurrence of isolated extrapulmonary TB

Variable	*n* (%)	*y* (%)	Univariate analysis OR (CI, *p* value)	Multivariate analysis OR (CI, *p* value)
WHO region				
European Region (EUR)	85 (71.4)	34 (28.6)	–	–
African Region (AFR)	50 (53.2)	44 (46.8)	2.20 (1.25–3.90, *p* = 0.006)	2.07 (1.15–3.78, *p* = 0.017)
Eastern Mediterranean Region (EMR)	37 (44.0)	47 (56.0)	3.18 (1.78–5.76, *p* < 0.001)	2.47 (1.36–4.57, *p* = 0.003)
Region of the Americas (AMR)	1 (33.3)	2 (66.7)	5.00 (0.46–109.69, *p* = 0.195)	3.46 (0.32–76.10, *p* = 0.319)
South-East Asian Region (SEAR)	23 (42.6)	31 (57.4)	3.37 (1.74–6.66, *p* < 0.001)	2.65 (1.33–5.35, *p* = 0.006)
Western Pacific Region (WPR)	7 (70.0)	3 (30.0)	1.07 (0.22–4.10, *p* = 0.924)	0.82 (0.17–3.22, *p* = 0.785)
HIV				
Yes	36 (81.8)	8 (18.2)	0.24 (0.10–0.51, *p* < 0.001)	0.25 (0.10–0.55, *p* = 0.001)
Immunosuppressive				
Yes	28 (87.5)	4 (12.5)	0.16 (0.05–0.42, *p* = 0.001)	0.20 (0.06–0.54, *p* = 0.004)
Age				
Mean (SD)	39.0 (15.6)	38.8 (15.8)	1.00 (0.99–1.01, *p* = 0.865)	–

## Discussion

In this study, we demonstrate that different geographical origins are associated with different clinical manifestations in TB.

In Germany, a majority of TB cases are observed in patients born outside the country, while the incidence in the autochthonous population is constantly decreasing. This is reflected in our study population, in which 309 patients (81.7%) were born abroad. Overall, we show a very low case fatality rate of 3.4%. In comparison to other mycobacterial infections, such as infections with non-tuberculous mycobacteria (NTM), this is an indicator for the excellent treatment options of TB in a high resource setting.

Origin from three WHO regions (SEAR, AFR and EMR) and especially seven countries (India, Pakistan, Nepal Afghanistan, Eritrea, Somalia, Turkey) was shown to be significantly associated with the occurrence of isolated extrapulmonary TB, while a majority of patients in our cohort originated from the Horn of Africa (Eritrea, Somalia, and Ethiopia) or the Indian Subcontinent (India, Nepal, Bangladesh, Pakistan, and Sri Lanka). However, only WHO regions with ten or fewer subjects did not qualify as being significantly associated with isolated EPTB. Nevertheless, our results are in line with health claims data on a European scale: Sotgiu et al. have shown that provenance from Africa or the Indian Subcontinent was significantly associated with the occurrence of extrapulmonary TB, while Hayward observed an association with origin from South-East Asia and sub-Saharan Africa [8, 9].

These differences might be linked to a delay in diagnosis as primary affection of the lung might not be treated in the respective home countries of patients born abroad. In addition, constraints that arise during a strenuous travel to Germany and socioeconomic factors might contribute to this effect. However, most patients were living for more than 3 years in Germany and most were living in their own flats or housings. Interestingly, patients from the seven countries associated with the occurrence of isolated extrapulmonary TB were significantly younger and suffered less frequently from relevant comorbidities. The fact that patients from Germany received immunosuppressive therapies more frequently and were suffering more frequently from HIV might be another explanation four our findings: these factors were shown to be negative predictors for the occurrence of isolated extrapulmonary TB and were positively associated with pulmonary TB (alone or with extrapulmonary foci). This might be linked to easier dissemination of the disease in immunocompromised hosts. Therefore, the geographical differences observed in our study might be partially explained by this effect. Amirkhani et al. have demonstrated that HIV infection was negatively associated with EPTB in Ethiopia, as well [15]. However, Khalife et al. for example describe a predominance of extrapulmonary TB in HIV-positive patients in Ukraine [16]. Besides obvious impairments of the immune system, such as HIV and medical immunosuppressive therapy, there might be susceptibility factors within a specific host attributable to geographic origin. Hypothetically, differences between different populations in the interferon gamma pathway and therefore the immune response to mycobacterial infection might be responsible for the variable clinical manifestations. In addition, different mycobacterial lineages have been shown to be associated with geographical origin [17]. This could be another factor explaining for different clinical manifestations. However, our analysis showed that other factors such as HIV infection and immunosuppressive therapies are unevenly distributed among the study population.

This study has several limitations: first, it is a monocentric study; second, we could not provide typing or whole genome sequencing data of bacterial isolates to be correlated with geographic origin; third, although for a single center case numbers are considerable, bigger study populations would be needed to underscore the shown effects.

In conclusion, we show that isolated extrapulmonary TB is more frequent in patients from India, Nepal, Pakistan, Afghanistan, Eritrea, Somalia, and Turkey at our tertiary care center. This observation gives a hint that geographical origin is a predictor for different host responses to MTB and confirms prior health claims data on a European scale. Specific impairments of the host’s immune system, possible virulence factors of the bacterium, as well as a delay in diagnosis and relevant comorbidities contributing to this effect will have to be investigated in future studies.

## Supplementary Information

Below is the link to the electronic supplementary material.

Supplementary file1 Figure S1: Flowchart of patient inclusion. (SVG 44 KB)

## Abbreviations

AMR: Region of the Americas
AFR: African Region
DS-TB: Drug-susceptible TB
EPTB: Extrapulmonary TB
EMR: Eastern Mediterranean Region
EUR: European Region
ICD: International Classifications of Diseases
IQR: Interquartile range
MDR-TB: Multidrug-resistant TB
MTB: *Mycobacterium tuberculosis* complex
NTM: Non-tuberculous mycobacteria
PTB: Pulmonary TB
RR-TB: Rifampicin-resistant TB
SEAR: South-East Asian Region
TB: Tuberculosis
WHO: World Health Organization
WPR: Western Pacific Region
XDR-TB: Extensively drug-resistant TB
